# Torsional Behavior of Concrete-Filled Circular Steel Tubes Strengthened with CFRP

**DOI:** 10.3390/ma16216964

**Published:** 2023-10-30

**Authors:** Wang Qing-li, Zhang Hui-ying, Kuan Peng

**Affiliations:** 1 School of Civil Engineering, University of Science and Technology Liaoning, Anshan 114051, China; 2 School of Intelligent Manufacturing, Chengdu Technological University, Chengdu 610031, China

**Keywords:** circular CFRP-steel tube, in-filled concrete, torsional behavior, numerical study, torsional bearing capacity

## Abstract

In order to study the torsional performance of steel tube concrete after reinforcement with a carbon-fiber-reinforced polymer (CFRP), the mechanical properties of 18 specimens were studied from both experimental and finite element perspectives. The *T*-*θ* curve and *τ*-*γ* curve of the specimen were measured in the experiment, and the failure mode of the specimen was analyzed. Subsequently, a reasonable finite element model was established using ABAQUS software, and the variation in various parameters surrounding the performance of the specimen was analyzed. Based on the experimental and finite element results, a formula for calculating the bearing capacity of the specimen was established. According to both the experimental and numerical results, the torsional bearing capacity of C-CF-CFRP-ST, defined as the torque endured by the specimen with maximum shear strain, was determined to be 15,000 με, together with its corresponding calculation formula. After the test, it was demonstrated that the main components of the concrete-filled CFRP-steel tube composite material—the steel tube and the concrete—could be used as reusable resources.

## 1. Introduction

Concrete-filled CFRP-steel tubes are composite materials, and its main components—a steel tube and concrete—are reusable resources. In particular, in recent years, composite structures of concrete, steel, and FRP have been research hotspots [1]. The concrete-filled CFRP-steel tube examined in this study has frequently appeared as the main research object at international conferences.

Liu Y et al. [2] investigated the torsion tests of 16 circular concrete-filled (CFRP)-steel tubes. The results showed that the failure modes of the specimens, bonded with longitudinal CFRP and circumferential CFRP, are different. Shakir et al. studied the mechanical properties of ordinary aggregate-concrete-filled or recycled aggregate-concrete-filled CFRP-steel tubes under lateral impact [3]. Their research results show that the deformation of ordinary aggregate-concrete-filled steel tube and recycled aggregate-concrete-filled steel tube specimens is similar, and their resistance to impact is also equivalent. The overall deformation of ordinary aggregate-concrete-filled steel tubes and recycled aggregate-concrete-filled steel tubes wrapped with CFRP is reduced. Chen et al. studied the impact performance of FRP steel tube concrete [4], and the results showed that the impact performance of FRP steel tube concrete is influenced by the type of FRP and the thickness of the steel tube. Thick steel tubes absorb more impact energy, which can improve the stiffness of specimens. Therefore, specimens have high impact resistance and small deformation. The constraint effect of CFRP, with the same thickness, on the specimen is better than that of GFRP. Liu Lan et al. conducted numerical simulation research on the dynamic response of 11 CFRP circular steel tube concrete column specimens under explosive load using the finite element software ANSYS (ANSYS 2023 R2) [5]. The research results indicated that, compared to concrete-filled steel tube columns, the lateral stiffness and explosion resistance of circular CFRP concrete-filled steel tube columns were significantly improved [6,7,8]. Zhu et al. established a hysteresis model for FRP-reinforced thin-walled steel tube concrete columns based on the stress–strain relationship between the local buckling of FRP-constrained steel tubes and the establishment of a restoring force model for FRP-constrained steel tubes in order to study the seismic mechanism of FRP-reinforced thin-walled steel tube concrete [9]. Wang Jingfeng et al. established a numerical model for eccentric compression performance, using ABAQUS software (ABAQUS 2020-6.14) to study the eccentric compression performance of short, circular steel tube concrete columns with void defects wrapped in CFRP spacing. A systematic parameter analysis was conducted on the eccentric compression of short columns of circular steel tube concrete with uniform void and spherical crown void wrapped in CFRP spacing [10]. As a new material, CFRP has lots of advantages, such as its high tensile strength, light weight, and so on. There are few studies on its performance, especially on complex forces such as torsion.

Therefore, an experimental and numerical study was conducted on circular CF-CFRP-ST (C-CF-CFRP-ST) specimens loaded with torsional moments. A total of 18 specimens, including 15 C-CF-CFRP-ST and 3 C-CFST specimens, were designed and tested. Numerical analysis was carried out for circular CF-CFRP-ST columns under torsion on the basis of the experimental results achieved. Then, a parametric study was conducted to explore the influence of principle factors, such as the CFPR layers, the strength of concrete and steel, and the steel ratio, on the torsional behavior of C-CF-CFRP-ST. Finally, an expression was proposed to estimate the torsional bearing capacity of C-CF-CFRP-ST based on the experimental and numerical analysis results.

## 2. Experimental Study

### 2.1. Material Properties of Specimens

The torsional performance tests of 15 circular CFRP CFSTs and 3 circular CFSTs were studied. The main parameters included *m*_t_, *m*_1_, and *f*_cu_. According to the requirements of the Technical Specification for CFST (GB 50936-2014) [11], the length *L* of all test pieces was 360 mm, the outer diameter *D*_s_ of the steel tube was 120 mm, and the thickness *t*_s_ of the steel tube was 2 mm. The other parameters of the specimens are shown in Table 1.

*f*_y_, *f*_u_, *E*_s_, *v*_s_ and *ε*′ of steel used for specimens are shown in Table 2.

The specific ratio of the concrete is shown in Table 3.

Finally, the cube compressive strengths *f*_cu_ of class A, B, and C concrete for 28 d are 35.1 MPa, 46.1 MPa, and 54.9 MPa respectively, and the elastic moduli *E*_c_ are 31.5 GPa, 33 GPa, and 35.2 GPa respectively.

The carbon fiber used was woven by the Japanese company Toray. The main performance indices of carbon fiber are shown in Table 4.

### 2.2. Loading and Measurement

**Figure 1:**
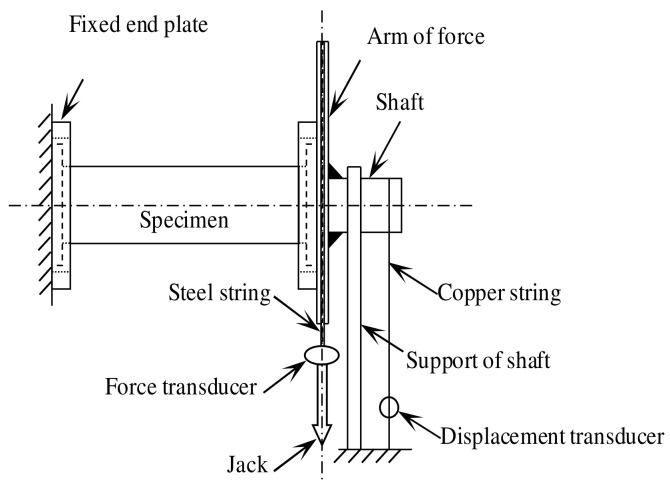
Loading equipment of C-CF-CFRP-ST specimen.

The loading diagram of the C-CF-CFRP-ST specimen is shown in Figure 1.

**Figure 2:**
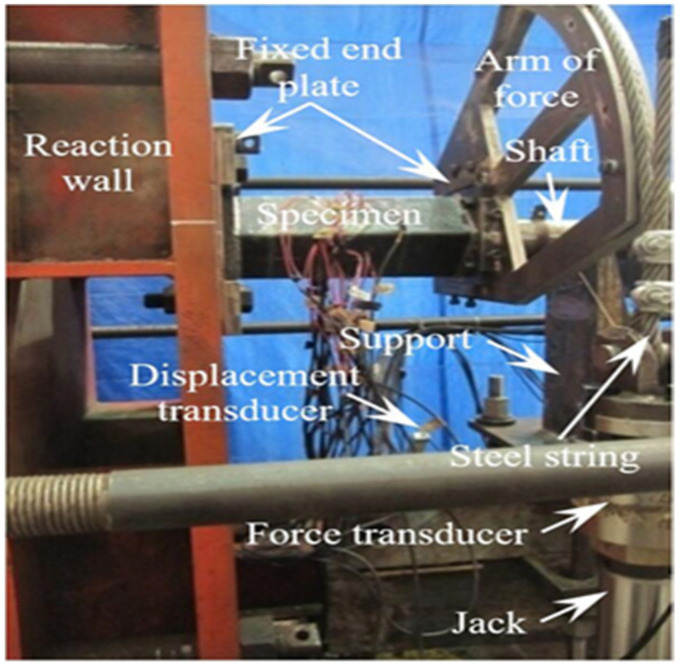
Loading panorama of specimen.

Figure 2 is the loading panorama of the C-CF-CFRP-ST specimen.

**Figure 3:**
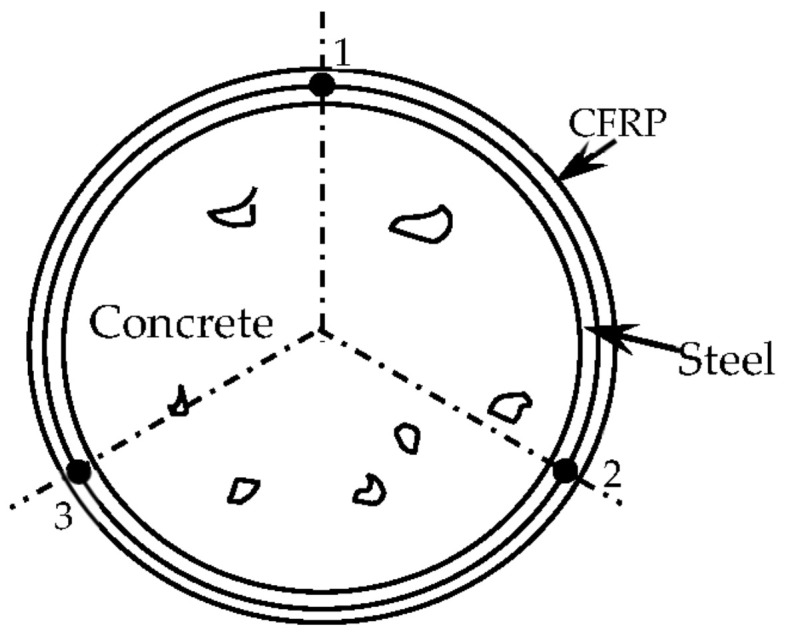
Arrangement of strain gauges for C-CF-CFRP-ST specimens.

The rotation angle is converted via the linear displacement generated via the tension of the displacement meter connected to the rigid rotating shaft. As shown in Figure 3, six resistance strain gauges are arranged at points 1–3 separated by 120° on the steel tube and CFRP in the middle section of the circular specimen to measure *ε*_t_, *ε*_l_, and *ε*_45_.

### 2.3. Experimental Phenomenon

**Figure 4:**
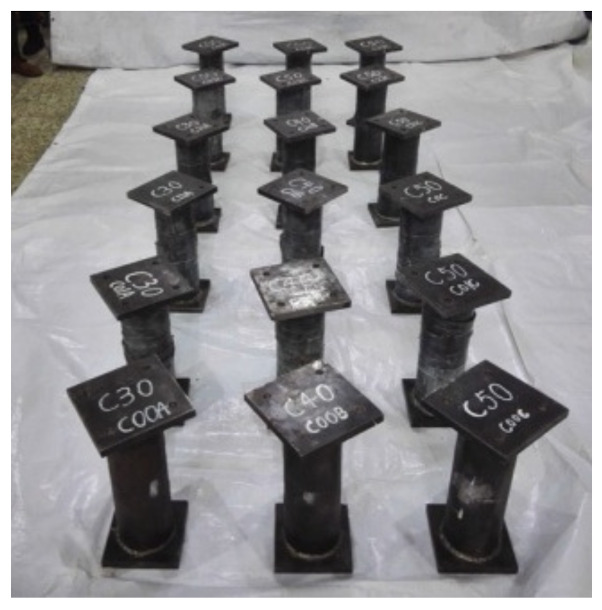
All specimens after loading.

**Figure 5:**
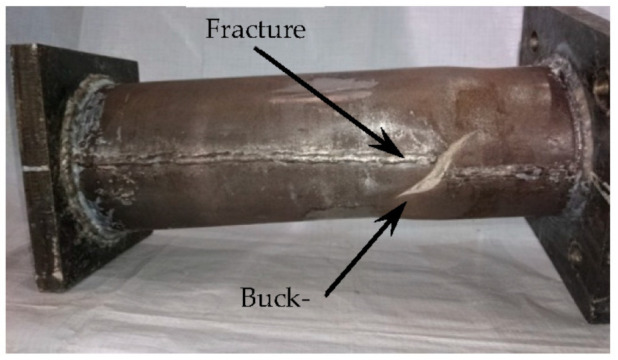
Failure of steel tube after loading.

**Figure 6:**
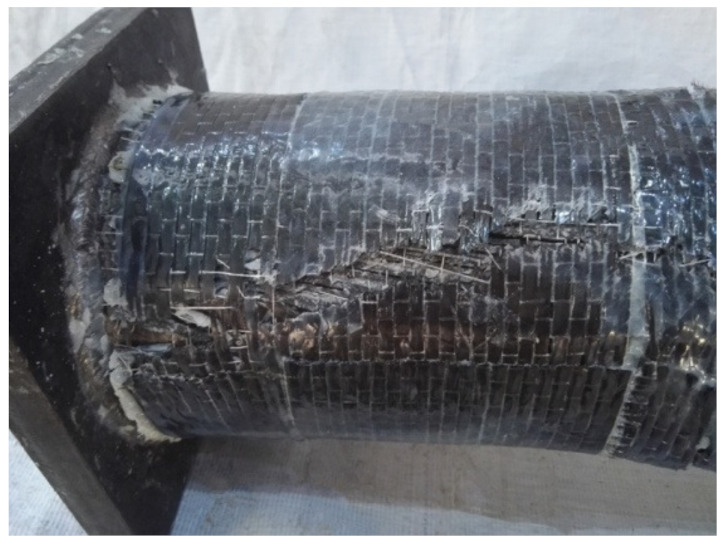
Transverse CFRP fracture.

In the initial loading stage, the linear relationship between the displacement and torque is reached, and the torsional deformation of the specimen can hardly be observed. When the torque is continuously applied, the transverse CFRP begins to fracture, the torque decreases to a certain extent, and then the longitudinal CFRP begins to fracture. After large-area fracture of CFRP, the torque still increases slightly, and the specimens show good ductility. In the later stage of loading, the specimens are pulled and cracked. For specimens without the longitudinal CFRP or with fewer longitudinal CFRP layers than the transverse CFRP, the load does not decrease significantly when the transverse CFRP breaks. All test pieces after loading are shown in Figure 4. The typical failure mode of the concrete-filled circular steel tube specimen is shown in Figure 5. The transverse CFRP fracture is shown in Figure 6. It can be seen that the transverse CFRP exhibits oblique cracking, indicating that the specimen is greatly affected by torsion, thus causing this type of damage to the CFRP.

**Figure 7:**
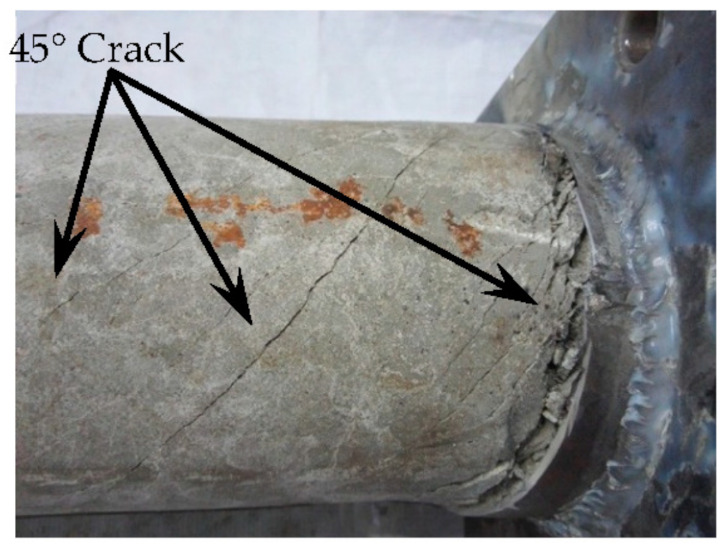
Concrete failure.

After loading, the steel tube was cut. It can be seen that there are many cracks in the concrete, and they do not penetrate the section. This shows that at the initial stage of loading, the concrete and the outer tube work together, while at the later stage of loading, the concrete and the outer tube are separated, and relative dislocation occurs between them. The damage to the concrete is shown in Figure 7.

### 2.4. Analysis of Test Results

#### 2.4.1. Moment–Rotation Curve

**Figure 8:**
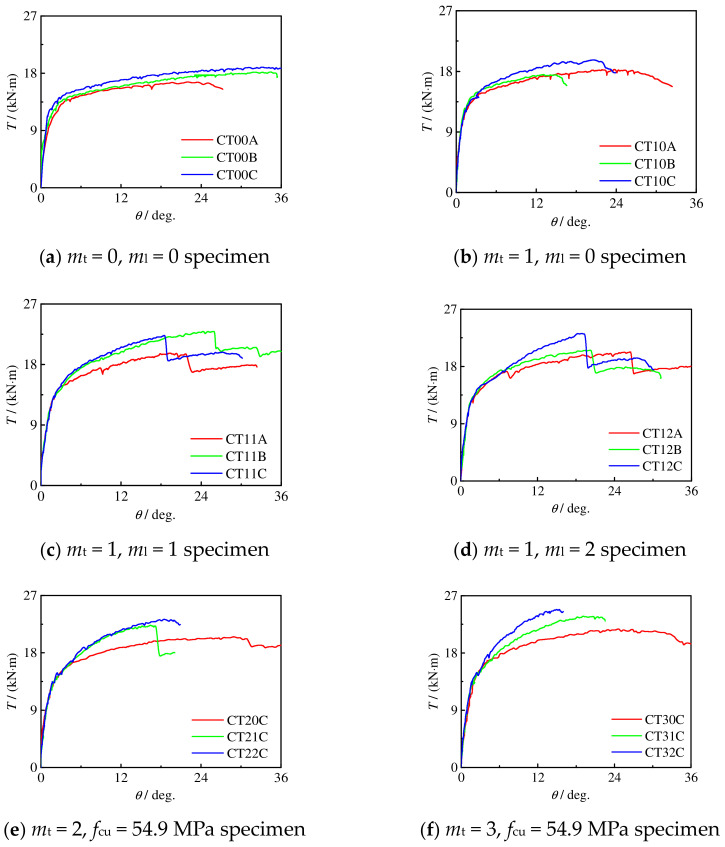
*T*-*θ* curve of all specimens.

Figure 8 shows the measured *T*-*θ* curve of the specimen. The curve is roughly divided into four stages: in the first elastic section, the steel tube and concrete resist torque together, and the stiffness is large; in the second elastic section (from the concrete cracking to the steel entering the elastic–plastic stage), the tensile cracking of concrete gradually exits the work, and the stiffness is lower than that in the first stage; in the third stage (steel yielding to transverse CFRP fracture), the specimen deformation increases rapidly, and the torque increases slowly; and in the fourth stage, the transverse CFRP breaks until the specimen is damaged. With the increase in torque, the longitudinal CFRP also breaks successively. The specimen after CFRP fracture is equivalent to the concrete-filled steel tubular specimen.

For C-CF-CFRP-ST specimens, when *m*_t_ is certain and *m*_t_ ≤ 2 and *m*_l_ ≥ 1, there is a large steep drop section in the *T*-*θ* curve of the specimens, and the corresponding angle decreases with the increase in *m*_l_. The reasons are as follows: when the transverse CFRP breaks, the longitudinal CFRP immediately peels off from the steel tube, accelerating the fracture of the transverse CFRP; the higher the number of layers of longitudinal CFRP, the easier it is to peel off, and the smaller the corresponding angular displacement during a steep drop. When *m*_l_ is constant and *m*_l_ ≠ 0, with the increase in *m*_t_, the steep falling section of the *T*-*θ* curve is less as well as less obvious. When the number of transverse CFRP layers is small, the effect of the longitudinal CFRP is more significant than that of the transverse CFRP. With the fracture of the transverse CFRP, the longitudinal CFRP immediately peels off, and the *T*-*θ* curve of the specimen has an obvious steep drop section. When there are many transverse CFRP layers, because the CFRP is locally fractured, the transverse CFRP at the non-fractured part still has a strong constraint on the longitudinal CFRP.

#### 2.4.2. Shear Stress–Strain Curve

**Figure 9:**
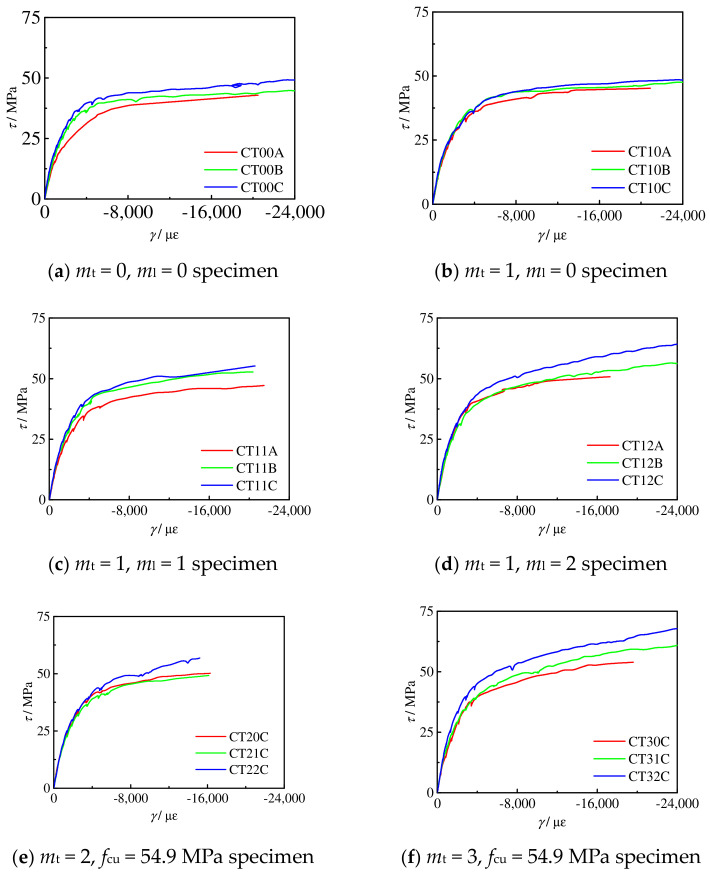
*τ*-*γ* curve of all specimens.

Figure 9 shows the measured shear stress–shear strain (*τ*-*γ*) curves of the circular concrete-filled CFRP-steel tube torsional specimens.

*τ* = *T*/*W*_sct_ (1)

*γ* = 2*ε* − −(*ε*_l_ + *ε*_t_) (2)

where *W*_sct_ is the torsional section modulus.

Using Formula (2), strain can be calculated for the circular CFRP concrete-filled steel tube. It can be seen that the law of the *τ*-*γ* curve is basically consistent with that of the *T*-*θ* curve.

#### 2.4.3. Collaborative Work of Steel Tube and CFRP

**Figure 10:**
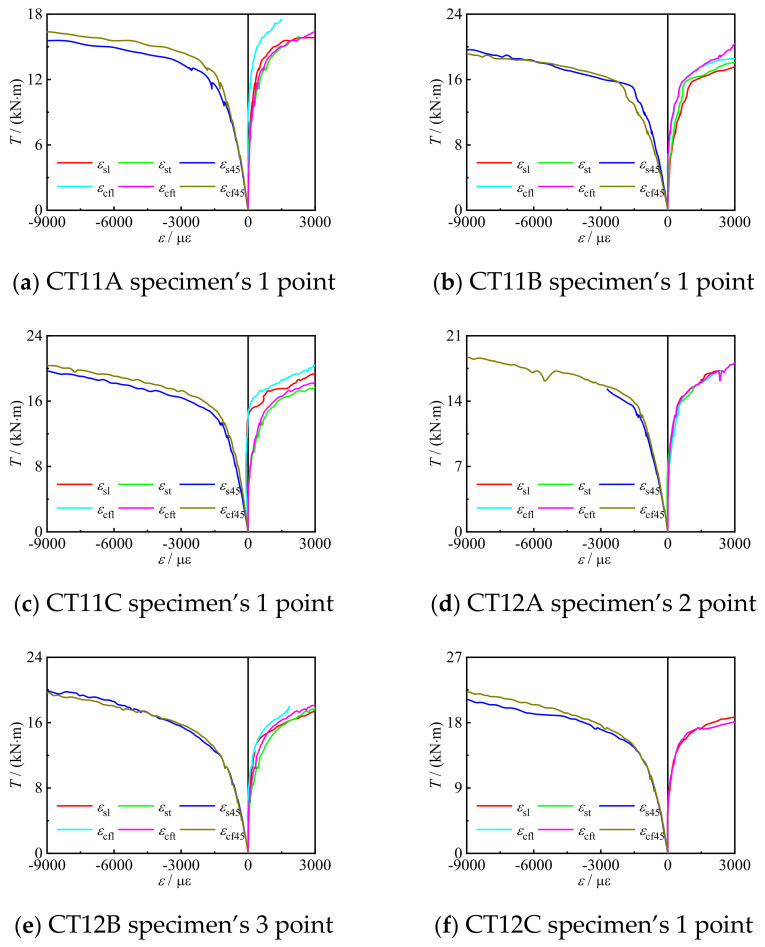
*T*-*ε* curve.

Figure 10 shows the *T*-*ε* curves of the specimens. It can be seen that *ε*_cf_ and *ε*_s_ are basically the same, which indicates that the two materials can work together. In addition, both *ε*_l_ and *ε*_t_ are positive, while *ε*_45_ is negative.

#### 2.4.4. Plane Section Assumption

**Figure 11:**
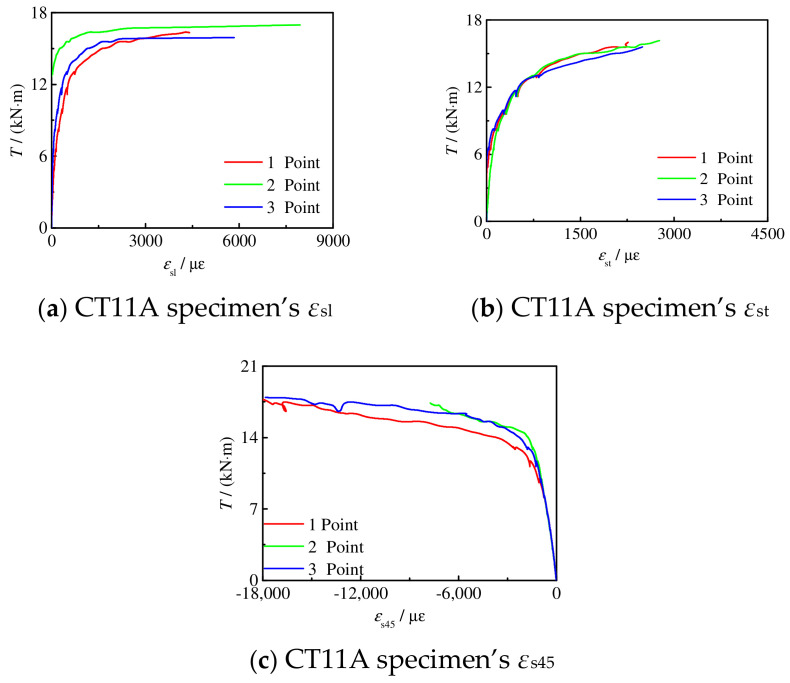
*T*-*ε*_s_ curve of C-CF-CFRP-ST specimen.

Figure 11 shows the performance of the *T*-*ε*_s_ curve of the partial C-CF-CFRP-ST specimens, where *ε*_sl_, *ε*_st_, and *ε*_s45_ represent the longitudinal strain, transverse strain, and strain in the 45° direction of the steel tube, respectively, and *ε*_cfl_, *ε*_cft_, and *ε*_cf45_ represent the longitudinal strain, transverse strain, and strain in the 45° direction of CFRP, respectively.

## 3. FE Model

### 3.1. Finite Element Calculation Model

**Figure 12:**
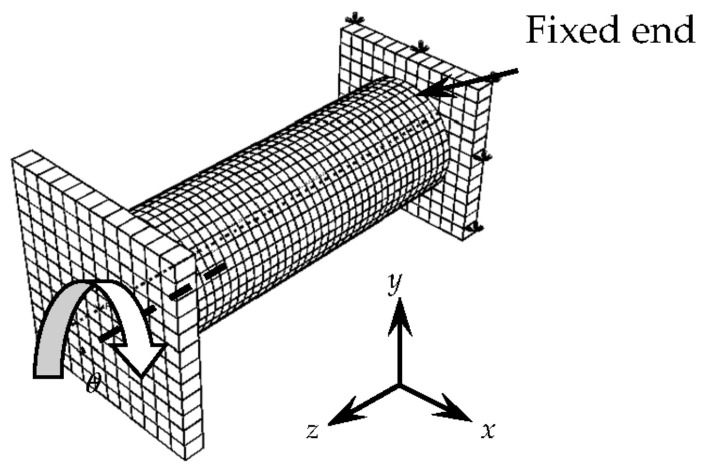
Boundary conditions for finite element simulation of C-CF-CFRP-ST specimens.

The finite element models (FEMs), consisting of concrete, square steel tube, CFRP, and end plate, were built according to the dimensions of the tested specimens, as described in Figure 12. The environmental conditions (humidity and temperature) are not relevant to the research results and model adaptation. The mesh specification and size are shown in Table 5. The mesh around the corners of the specimens is refined considering the corner effect, and the mesh convergence of FEM is inspected before analysis [12,13,14].

### 3.2. Comparison between Simulation Results and Test Results

#### 3.2.1. *T*-*θ* Curve

**Figure 13:**
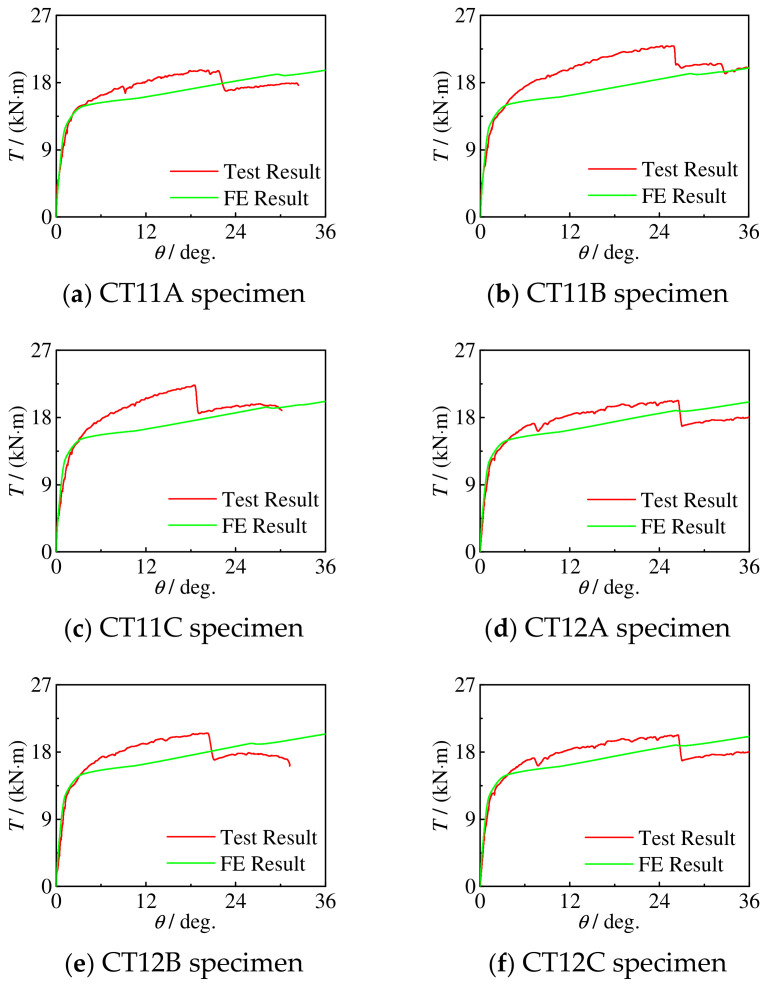
Comparison of *T*-*θ* curve simulation results and test results of C-CF-CFRP-ST specimens.

Figure 13 shows a comparison of the *T*-*θ* curve simulation results and the test results of the performance of the C-CF-CFRP-ST specimens. It can be seen that the simulation results are in good agreement with the experimental results.

#### 3.2.2. Failure Mode

**Figure 14:**
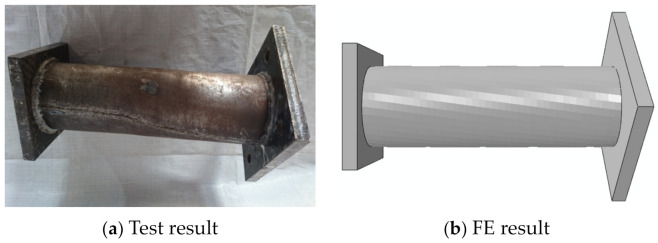
Failure mode of steel tube.

**Figure 15:**
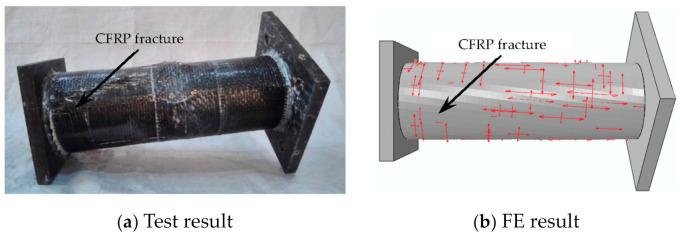
Failure mode of transverse CFRP.

**Figure 16:**
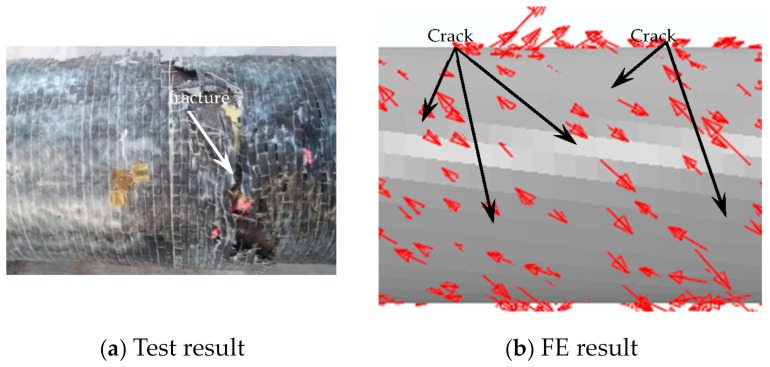
Failure mode of longitudinal CFRP.

**Figure 17:**
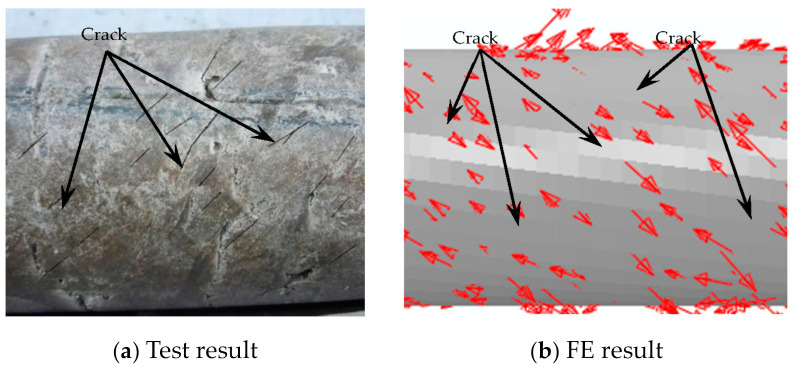
Failure mode of concrete.

In this section, in order to more clearly verify the rationality of the finite element model, the failure mode diagrams of the experiment and finite element are compared separately. Figure 14, Figure 15, Figure 16 and Figure 17 show the failure modes of the main constituent materials of the C-CFST component. Based on this, the rationality of the proposed numerical simulation method for the torsional performance of C-CF-CFRP-ST specimens is verified.

## 4. Analysis of the Whole Process of Stress

**Figure 18:**
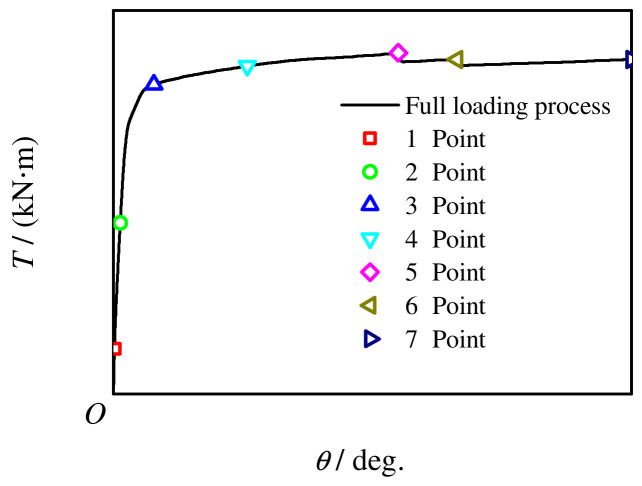
Loading curve of typical specimen.

Figure 18 shows the typical *T*-*θ* curve of the C-CF-CFRP-ST specimens. Because the concrete has micro-cracks and some concrete breaks, points 1–2 are the elastic stage of stiffness degradation. After this, the torque is mainly borne by the outer CFRP steel tube, but the inner concrete plays a good filling role, avoiding the buckling of the outer tube and ensuring that the steel tube and CFRP can fully leverage their respective strengths. At this time, the transverse CFRP and the longitudinal CFRP are not broken, the rotation angle of the specimen is about to increase sharply, and the *T*-*θ* curve is relatively flat. Torque corresponding to point 4 is defined as the torsional bearing capacity, point 5 corresponds to transverse CFRP fracture, point 6 corresponds to longitudinal CFRP fracture, and point 7 corresponds to 45° [15,16,17].

The following are the calculation parameters: *L* = 360 mm, *D*_s_ = 120 mm, *t*_s_ = 2.0 mm, *f*_cu_ = 60 MPa (standard value of concrete axial compressive strength, *f*_ck_ = 40 MPa), *E*_c_ = 4700 *f* ′_c_^0.5^ (*f* ′_c_ is the concrete cylinder compressive strength, 44.8 MPa), *f*_y_ = 345 MPa, *ξ*_s_ = 0.605, *ξ*_cf_ = 0.125, and *η* = 0.159.

### 4.1. Stress Distribution of Steel Tube and Concrete

**Figure 19:**
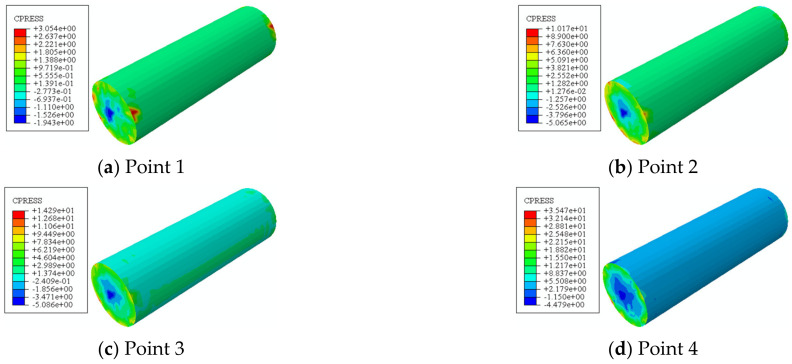

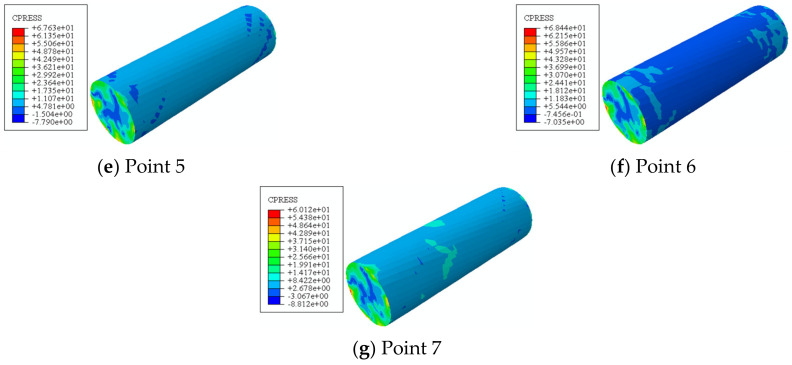
Stress distribution of steel tube and concrete.

Figure 19 shows the stress distribution of the outer tube and concrete. At point 1, the stress is small because the deformation of the specimen is still very small. Subsequently, the deformation of the specimen increases gradually, the concrete crack develops gradually, the transverse deformation rate is higher than that of the steel tube, and the interaction force between them increases gradually. In the later stages of loading, the force decreases.

### 4.2. Concrete Stress

**Figure 20:**
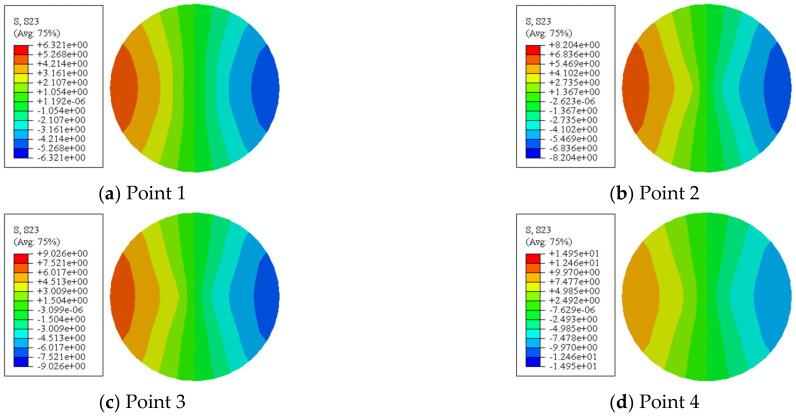

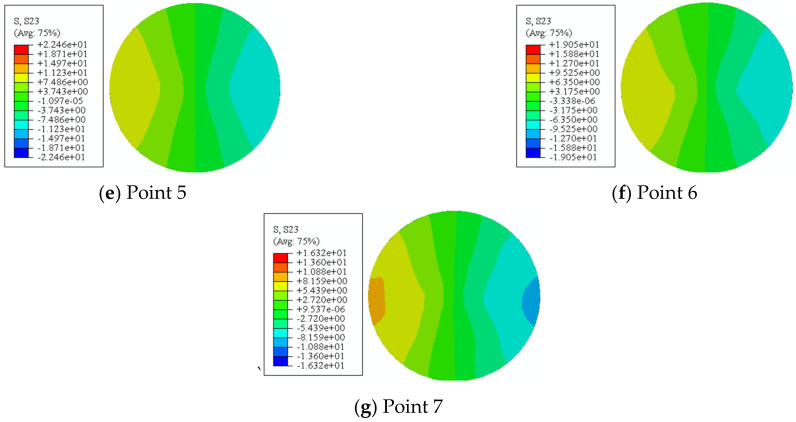
Shear-stress distribution of concrete in section of C-CF-CFRP-ST specimen.

The stress distribution of the concrete is shown in Figure 20. At point 1, the maximum shear stress of the specimen reaches 6.321 MPa, and the shear stress from this point to the corner and to the shape mandrel gradually decreases. In the subsequent loading process, the distribution of shear stress on the whole cross-section of circular specimens tends to be uniform, and the stress contour is more and more “V”-shaped.

### 4.3. Steel Tube Stress

**Figure 21:**
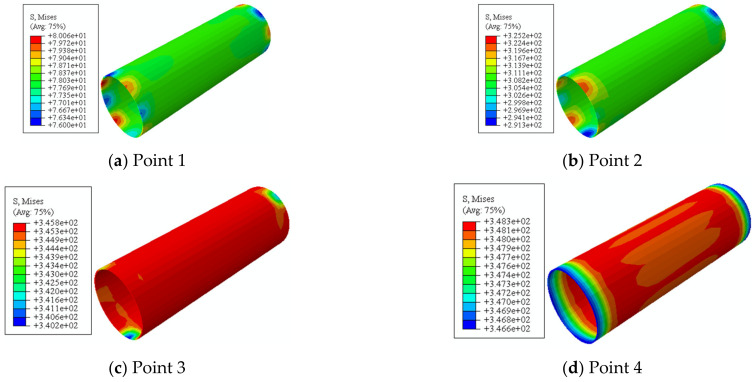

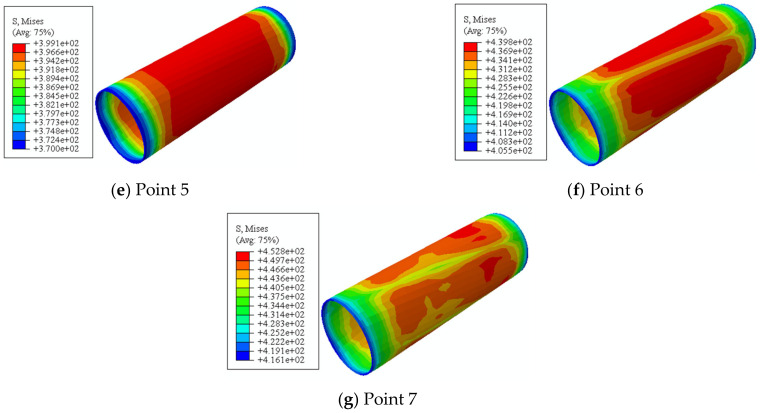
Mises stress distribution of C-CF-CFRP-ST specimen.

Figure 21 shows the Mises stress distribution. From point 1 to point 3, the steel pipe gradually moves from the elastic stage to the yield stage, and the stress significantly increases. At point 3, the stress of the circular component reaches 345 MPa. Subsequently, the steel pipe enters the stage of plastic reinforcement, and the stress continues to increase. The Mises stress of the component is always uniformly distributed.

### 4.4. CFRP Stress

**Figure 22:**
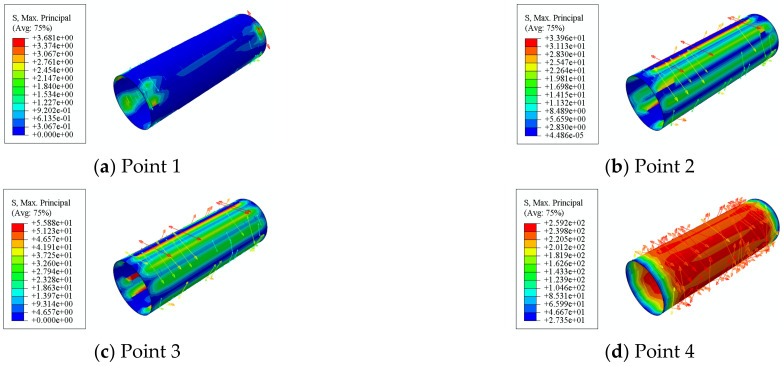

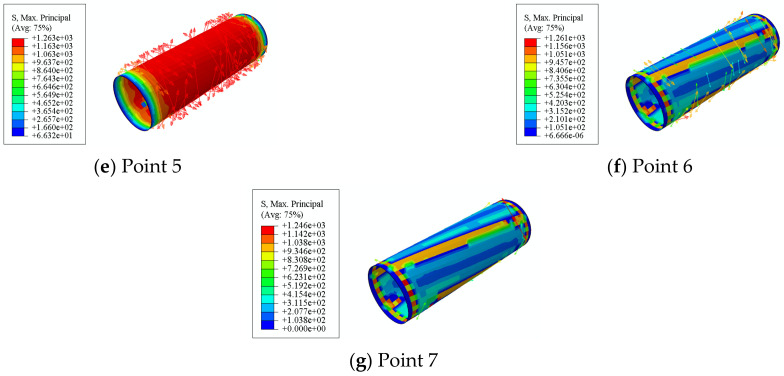
Transverse CFRP stress distribution.

Figure 22 shows the transverse CFRP stress distribution of the C-CF-CFRP-ST specimens. In the initial stage of loading, there is basically no significant deformation in the component. As load continues to be applied, the stress of CFRP gradually increases. When loaded to point 5, the transverse CFRP stress of the specimen reaches 1263 MPa. After this, the transverse CFRP begins to fracture over a large area, and the stress at the fracture position tends to be zero. This shows that the transverse CFRP has a good restraining effect on the specimens.

## 5. Parameter Analysis

The influence laws of the above parameters are analyzed through calculation examples (*L* = 360 mm, *D*_s_ = 120 mm, *t*_s_ = 2.0 mm, *f*_cu_ = 60 MPa, *E*_c_ = 4700 *f* ′_c_^0.5^(*f* ′_c_ = 44.8 MPa), *f*_y_ = 345 MPa, *α* = 0.07, *ξ*_cf_ = 0.125, and *η* = 0.159).

### 5.1. Influence of CFRP Layers

**Figure 23:**
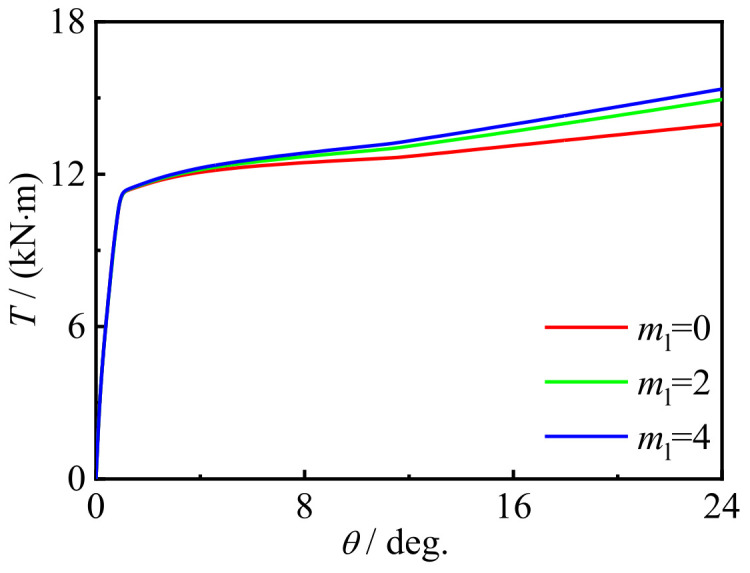
*m*_l_ effect on the *T-θ* curve of the specimens.

**Figure 24:**
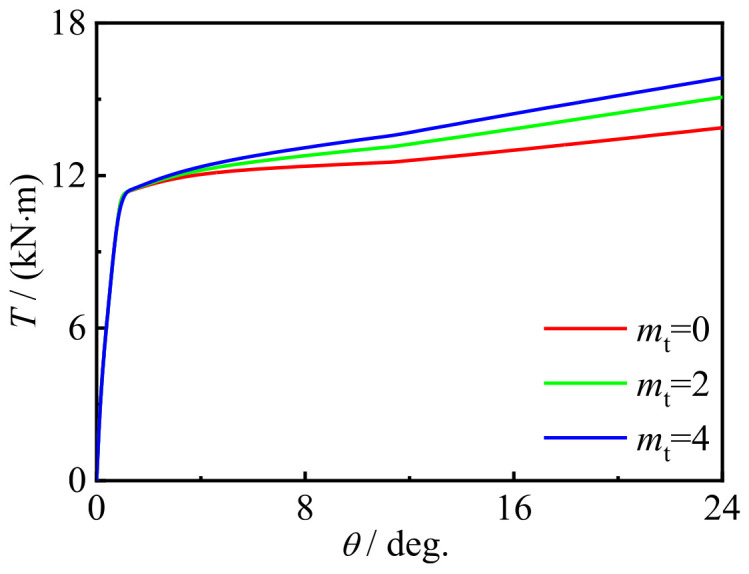
*m*_t_ effect on the *T-θ* curve of the specimens.

Figure 23 and Figure 24 show the effect of the number of CFRP layers on the performance changes in the specimen. Due to the good restraining effect, the bearing capacity slightly increases with the increase in the number of CFRP layers.

### 5.2. Effect of Material Strength

**Figure 25:**
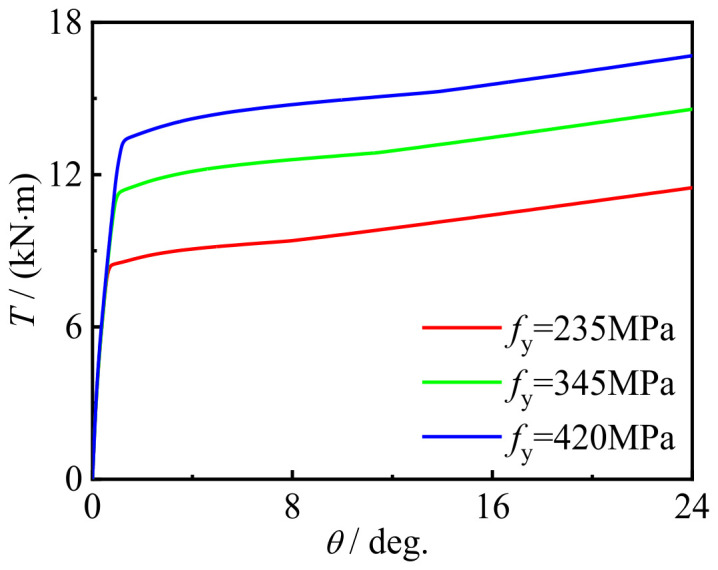
*f*_y_ effect on the *T-θ* curve of C-CF-CFRP-ST specimens.

**Figure 26:**
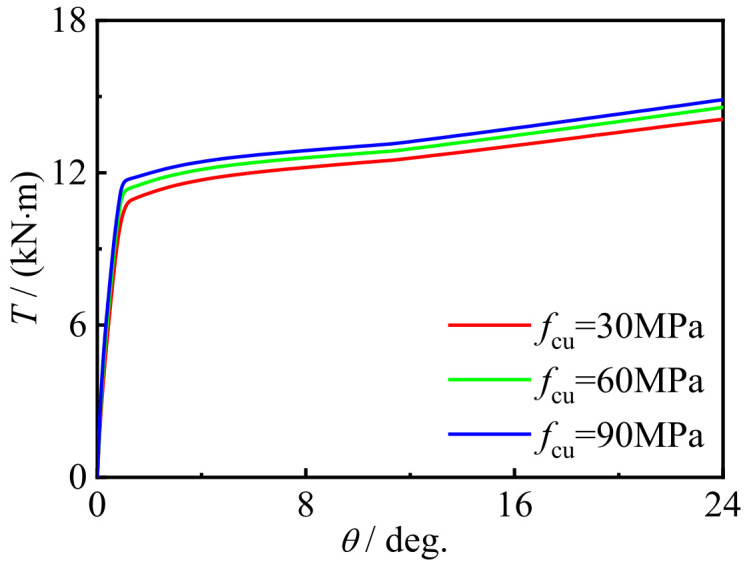
*f*_u_ effect on the *T-θ* curve of C-CF-CFRP-ST specimens.

Figure 25 and Figure 26 show the effects of steel yield strength and concrete strength on the performance of the torque angle (*T*-*θ*) curve of the C-CF-CFRP-ST specimens. With the improvement in the strength of the concrete and steel materials, the bearing capacity of the specimens is improved, and the influence of steel is more obvious.

### 5.3. Effect of Steel Content

**Figure 27:**
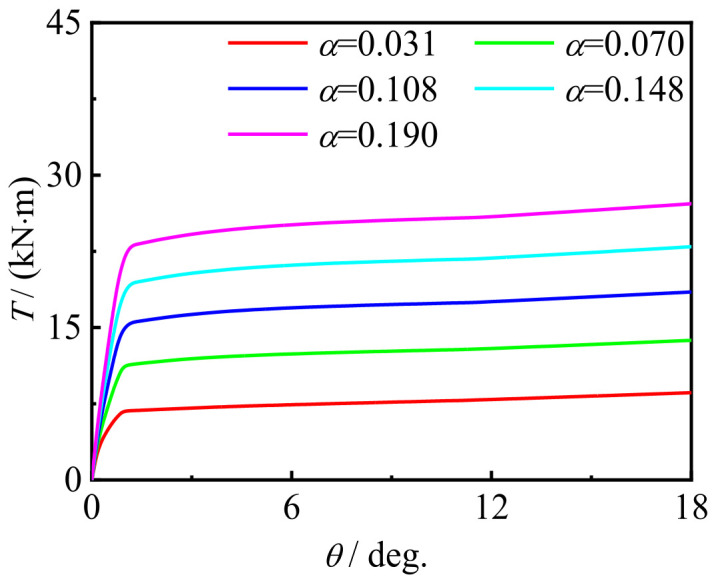
*α* effect on the *T*-*θ* curve of C-CF-CFRP-ST specimens.

Figure 27 shows the effect of steel content. It can be seen that with the increase in (steel content) *α*, the curve shape barely changes, but the initial stiffness and bearing capacity increase significantly.

## 6. Torsional Bearing Capacity

### 6.1. Definition of Torsional Capacity

A reasonable definition of torsional strength has practical significance. If the torsional strength is defined as too small, the component performance cannot be leveraged, which will lead to problems such as material waste. If the torsional strength is defined as too large, the safety reserve of the components is not enough, which may lead to engineering quality problems [18]. The torque corresponding to the maximum shear strain of a specimen is defined as 10,000 με as the torsional bearing capacity of concrete-filled steel tubular. Through the sorting and analysis of the test phenomena and data, this study defines the torque corresponding to the maximum shear strain of a specimen up to 15,000 με as the torsional bearing capacity of the specimen.

### 6.2. Calculation Expression

Applicable scope: the length–diameter ratio is 3, *f*_y_ = 235 MPa~460 MPa, *f*_cu_ = 30 MPa~90 MPa, *α* = 0.05~0.2, *ξ*_s_ = 0.2~4, *ξ*_cf_ = 0~0.6, and *η* = 0~1. The relationship between the torsional strength *τ*_cfscy_ and the above parameters is regressed:

(3) $$\tau_{\mathrm{cfscy}}=e^{-1.5\xi_{\mathrm{cf}}}\left(0.6+0.1\eta +0.313\alpha^{2.33}\right)\xi^{\left(0.134+1.2\xi \prime \right)}f_{\mathrm{cfscy}}$$

where *f*_cfscy_ refers to the axial compressive strength of the CFRP concrete-filled steel tube.

The calculation expression of the torsional bearing capacity of CFRP concrete-filled steel tubular is as follows:

*T* = *τ*_cfscy_*W*_cfsct_ (4)

where *W*_cfsct_ represents torsional section modulus.

### 6.3. Validation of Expressions

**Figure 28:**
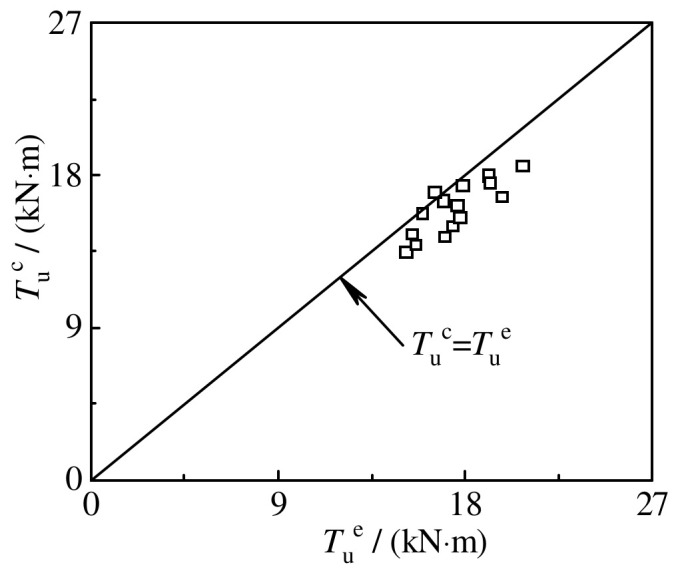
The comparison between the calculation result *T*_u_^c^ of torsional bearing capacity and the test result *T*_u_^e^.

Figure 28 shows the comparison between the calculation result *T*_u_^c^ of torsional bearing capacity and the test result *T*_u_^e^. The average value of *T*_u_^c^/*T*_u_^e^ of the circular specimens is 0.916, and the mean square deviation is 0.05.

In order to better introduce the conclusion section, all conclusions are classified and presented in Table 6.

## 7. Conclusions

Based on the results of this study, the following conclusions can be made:

1. The typical *T*-*θ* curve of the CFRP concrete-filled steel tubule is given. The curve is characterized by the elastic stage from the beginning of loading to the cracking of concrete. At this time, the torque is borne by the outer tube and concrete, and the *T*-*θ* curve at this stage is linear. When the concrete cracks appear, the specimen enters the elastic–plastic stage. At this time, with the continuous increase in torque, the concrete cracks gradually develop, the concrete continues to withdraw from its load-bearing function, the torsional stiffness gradually decreases, and most of the torque is borne by the steel tube. When most of the concrete no longer contributes to the structural load, the specimen enters the plastic reinforcement stage. Because of the restraint effect of the outer tube, the broken concrete is still whole, and the buckling of the steel tube is restrained. The steep drop in the *T*-*θ* curve is due to the fracture of the transverse and longitudinal carbon fibers, which also shows that CFRP CFST has stronger torsional resistance than CFST alone.
2. The *z*-axis component *τ*_yz_ of the shear stress of the concrete/steel tube in the middle section of the specimen is evenly distributed along the length direction of the specimen. The maximum tensile stress of the concrete in the middle section of the circular specimen increases from inside to outside along the section radius, and the maximum tensile stress is evenly distributed along the length direction of the specimen.
3. At the initial stage of loading, the interaction force between the steel tube and concrete is small. The interaction force between the steel tube and concrete is equal in the symmetrical position of the same section, and the interaction force between the steel tube and concrete is evenly distributed along the axis.
4. With the development of green buildings, it is becoming more and more important to determine whether constituent materials can be used as renewable resources after the structure reaches its service life. The concrete-filled CFRP-steel tube examined in this study is made of steel and concrete, and the steel tube and concrete in its main components could be used as reusable resources. The steel tube and concrete performance after tests will also be studied in the future.

## Figures and Tables

**Table 1:** Parameters of C-CF-CFRP-ST torsional specimens.

No.	Specimen Labels	*f*_cu_ (MPa)	*m*_t_ Layer(s)	*m*_l_ Layer(s)	*x*_cf_	*h*_cf_	*x*_s_
1	CT00A	31.5	0	0	1.38	0	0
2	CT00B	33	0	0	1.05	0	0
3	CT00C	35.2	0	0	0.88	0	0
4	CT10A	31.5	1	0	1.38	0.21	0
5	CT10B	33	1	0	1.05	0.16	0
6	CT10C	35.2	1	0	0.88	0.17	0
7	CT11A	31.5	1	1	1.38	0.21	0.2
8	CT11B	33	1	1	1.05	0.16	0.2
9	CT11C	35.2	1	1	0.88	0.17	0.2
10	CT12A	31.5	1	2	1.38	0.21	0.4
11	CT12B	33	1	2	1.05	0.16	0.4
12	CT12C	35.2	1	2	0.88	0.17	0.4
13	CT20C	35.2	2	0	0.88	0.27	0
14	CT21C	35.2	2	1	0.88	0.27	0.2
15	CT22C	35.2	2	2	0.88	0.27	0.4
16	CT30C	35.2	3	0	0.88	0.41	0
17	CT31C	35.2	3	1	0.88	0.41	0.2
18	CT32C	35.2	3	2	0.88	0.41	0.4

**Table 2:** Performance of steel tubes used in C-CF-CFRP-ST specimens.

*f*_y_/MPa	*f*_u_/MPa	*E*_s_/GPa	*v*_s_	*ε*′/%
466	610	206	0.28	27

**Table 3:** Mixed proportion of concrete used for C-CF-CFRP-ST specimens.

No.	C	FA	S	G	W	SP
A	0.6	0.4	2.5	1.5	0.4	0.01
B	0.6	0.4	2	1.4	0.35	0.01
C	0.74	0.26	1.2	1.5	0.3	0.009

**Table 4:** Property indices of CFRP.

Thickness (mm)	*E*_cf_ (GPa)	*e*_cftr_ (me)	*e*_cflr_ (me)
0.111	230	5500	7000

**Table 5:** Size and mesh type for specimens.

Geometry	Steel	Concrete	CFRP	End Plate
Section (mm)	120 × 120	98 × 98	Different dimensions	200 × 200
Thickness (mm)	2	/	Different dimensions	20
Specimen length (mm)	360	360	360	/
Type of geometry	Solid	Solid	Shell	Solid
Mesh type	C3D8R	C3D8R	M3D4	C3D8R

**Table 6:** Summary of experimental and finite element conclusions.

Type	Analytical
Test	1. The typical *T*-*θ* curve is characterized by the elastic stage from the beginning of loading to the cracking of concrete. 2. With the fracture of the transverse CFRP, the longitudinal CFRP immediately peels off, and the *T-θ* curve of the specimen has an obvious steep drop section.
Numerical studies	1. The simulation results are in good agreement with the experimental results. 2. At the initial stage of loading, the interaction force between the steel tube and concrete is small. The interaction force between the steel tube and concrete is equal in the symmetrical position of the same section, and the interaction force between the steel tube and concrete is evenly distributed along the axis.

## Data Availability

The datasets generated and/or analyzed during the study are available from the corresponding author upon reasonable request.
